# Transcutaneous vagus nerve stimulation (t-VNS): A novel effective treatment for temper outbursts in adults with Prader-Willi Syndrome indicated by results from a non-blind study

**DOI:** 10.1371/journal.pone.0223750

**Published:** 2019-12-03

**Authors:** Katherine E. Manning, Jessica A. Beresford-Webb, Lucie C. S. Aman, Howard A. Ring, Peter C. Watson, Stephen W. Porges, Chris Oliver, Sally R. Jennings, Anthony J. Holland

**Affiliations:** 1 Department of Psychiatry, University of Cambridge, Cambridge, Cambridgeshire, United Kingdom; 2 School of Health and Social Care, University of Essex, Colchester, Essex, United Kingdom; 3 Essex Partnership University NHS Foundation Trust, Wickford, Essex, United Kingdom; 4 MRC Cognition and Brain Sciences Unit, University of Cambridge, Cambridge, Cambridgeshire, United Kingdom; 5 Department of Psychiatry, University of North Carolina, Chapel Hill, North Carolina, United States of America; 6 School of Psychology, University of Birmingham, Birmingham, West Midlands, United Kingdom; Monash University, AUSTRALIA

## Abstract

Temper outbursts are a severe problem for people with Prader-Willi Syndrome (PWS). Previous reports indicate that vagus nerve stimulation (VNS) may reduce maladaptive behaviour in neurodevelopmental disorders, including PWS. We systematically investigated the effectiveness of transcutaneous VNS (t-VNS) in PWS. Using a non-blind single case repeat measures modified ABA design, with participants as their own controls, t-VNS was evaluated in five individuals with PWS [three males; age 22–41 (*M* = 26.8)]. After a baseline phase, participants received four-hours of t-VNS daily for 12 months, followed by one month of daily t-VNS for two-hours. The primary outcome measure was the mean number of behavioural outbursts per day. Secondary outcomes included findings from behavioural questionnaires and both qualitative and goal attainment interviews. Four of the five participants who completed the study exhibited a statistically significant reduction in number and severity of temper outbursts after approximately nine months of daily four-hour t-VNS. Subsequent two-hour daily t-VNS was associated with increased outbursts for all participants, two reaching significance. Questionnaire and interview data supported these findings, the latter indicating potential mechanisms of action. No serious safety issues were reported. t-VNS is an effective, novel and safe intervention for chronic temper outbursts in PWS. We propose these changes are mediated through vagal projections and their effects both centrally and on the functioning of the parasympathetic nervous system. These findings challenge our present biopsychosocial understanding of such behaviours suggesting that there is a single major mechanism that is modifiable using t-VNS. This intervention is potentially generalizable across other clinical groups. Future research should address the lack of a sham condition in this study along with the prevalence of high drop out rates, and the potential effects of different stimulation intensities, frequencies and pulse widths.

## Introduction

Prader-Willi Syndrome (PWS) is a genetically determined neurodevelopmental disorder with a birth incidence of one in 25,000–29,000.[1] At least 80% of people with PWS are reported to have severe temper outbursts from childhood into adult life.[2,3] These behaviours have a significant effect on the level of independence and quality of life of those with PWS and impact those who support them.[4]

PWS results from the absence of paternal expression of maternally imprinted genes at chromosomal locus 15q11-13, resulting from either a paternal interstitial deletion (delPWS; 70% of cases), maternal uniparental disomy (mUPD; 25%), or less commonly, an imprinting centre defect (3–5%) or an unbalanced translocation (<5%).[5] All genetic types of PWS are characterised by infantile hypotonia, hypogonadism, early failure to thrive, mild to moderate intellectual disability, distinct facial characteristics, short stature, and small extremities.[6] PWS also has a characteristic behavioural phenotype that includes a marked propensity to severe temper outbursts, the onset of severe hyperphagia in early childhood that results in life threatening obesity if food access is not controlled, repetitive and ritualistic behaviours, and skin picking.[2,3]

The reasons for chronic and lifelong temper outbursts in PWS are uncertain. The impact of developmental delay,[2] shaping of such behaviours through reinforcement, and a deficit in attention shifting, whereby unexpected changes in routine or expectations generate high cognitive demand, may all predispose to this high propensity for outbursts.[3,7–9] Presently, treatments are informed by a biopsychosocial perspective but often limited to the ad-hoc use of psychiatric medications and behavioural approaches that aim to reduce outburst frequency and severity, together with better management when they do occur. Given the adverse impact and the limits of present interventions the development of effective interventions is a high priority.

Recent unanticipated observations in a study of three adults with PWS[10] suggests a possible novel and effective intervention: vagus nerve stimulation (VNS). Using implanted devices, this study investigated whether VNS might reduce hyperphagia. Whilst no reduction in hyperphagia was found, improvements in problem behaviours were reported by two of the three participants who had a history of problem behaviours, and also by their carers. Anecdotally, these behavioural improvements had a notable effect on participants’ quality of life and the two participants asked to continue with the VNS activated after the end of the study and behaviour problems have remained very much improved over the last five years. Importantly, no significant side effects or safety issues have been reported.

VNS has been used primarily to treat epilepsy with reports indicating beneficial effects on behaviour and cognitive functioning, changes not explained by improved seizure control.[11,12] Similar beneficial effects of VNS have been demonstrated in people with neurodevelopmental disorders, including case studies reporting improvements in mood and reduction of aggressive outbursts in participants with ASD and epilepsy, independent of seizure control.[13–15]

The primary aim of this study was to investigate the efficacy of VNS using an externally worn stimulator (transcutaneous vagus nerve stimulation, t-VNS), as a therapy for temper outbursts in PWS. Secondary aims were: a) to study the acceptability and tolerance of t-VNS, and b) to investigate the profile of response and possible mechanisms in order to identify markers predictive of any change, so as to inform future trials and ultimately clinical practice. This paper reports the outcomes of the intervention. Underlying mechanisms of action are the subject of on-going investigation and will be reported in a subsequent paper.

## Methods

East of England–Cambridge Central Research Ethics Committee (15/EE/0450) granted ethical approval. The study was registered as a clinical trial (NCT03689621) retrospectively due to the ethics committee at the beginning of the study defining it as a ‘clinical investigation’ not ‘clinical trial’. To adhere to the ethics committee’s regulations, the study was registered on the publically accessible NIHR UK Clinical Research Network (registration number: CMPS20829). The authors confirm that all on-going and related trials for this intervention are registered.

A repeated measures modified ABA design case series using quantitative and qualitative outcome measures with participants as their own controls was undertaken. A randomised trial with a blinded condition was not possible as the recommendation for t-VNS use requires participants to set the stimulation level so as to experience a tingling sensation in the ear. A baseline period with treatment continuing as usual for a minimum of four months was followed by an active treatment phase of initially six months, later extended to 12 months. If no benefits were observed the study would cease at this point. In the event of observed improvements in behaviour ethical approval was given for a reduction in stimulation time by 50% to determine whether benefits would be maintained at this lower level. However, if temper outbursts returned during this phase, given their potential severity, it was considered unethical not to re-establish the stimulation time at the recommended level if the participants so requested. Participants who experienced improvements in behaviour were also given the option to continue t-VNS after study completion. During data collection participants lived in their usual places of residence.

### Participants

**Fig 1:**
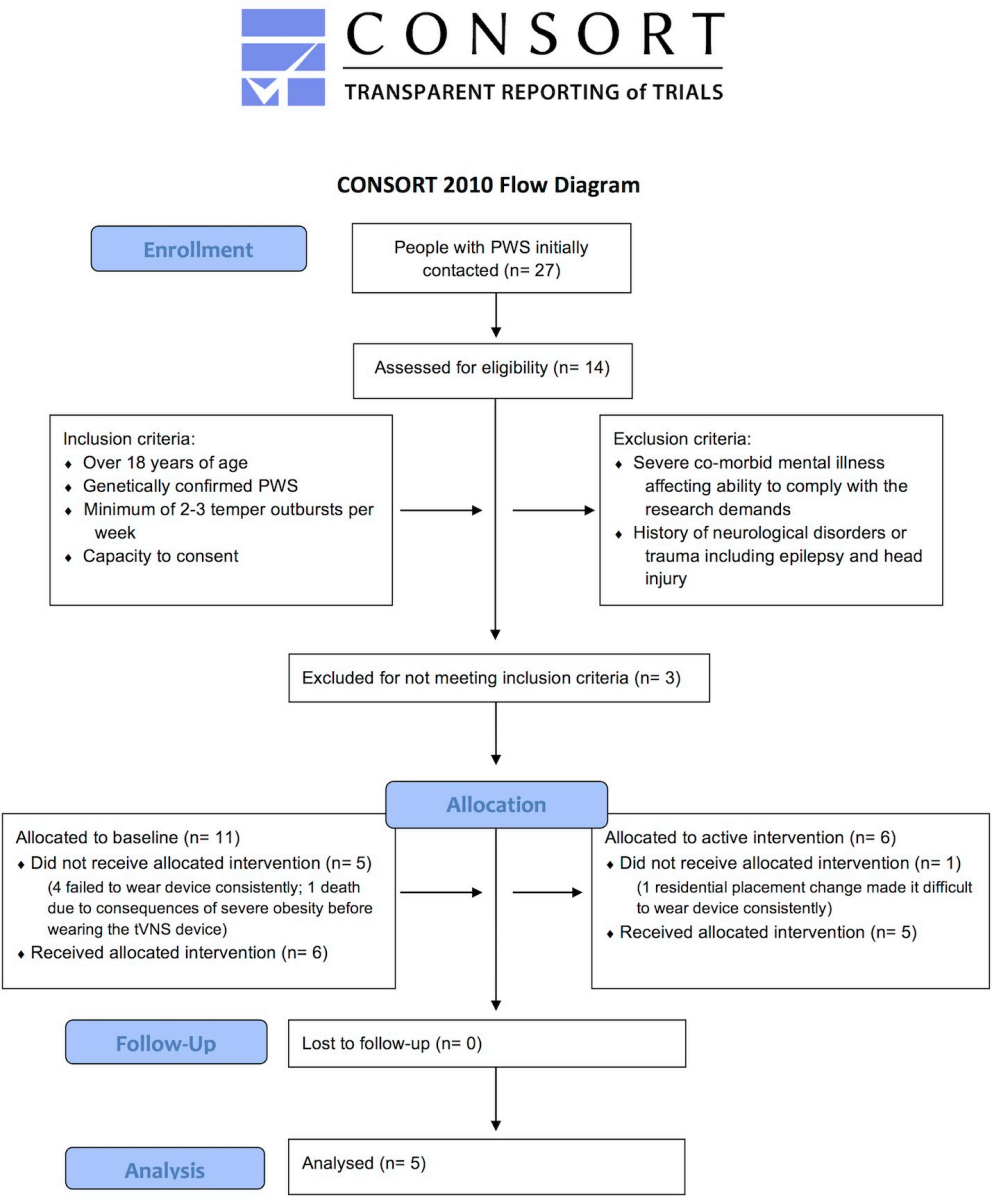
Consolidated Standards of Reporting Trials (CONSORT) flow chart. CONSORT flow chart illustrating all steps in the study from enrollment to allocation and follow up. Inclusion and exclusion criteria are also specified.

The inclusion and exclusion criteria and subsequent participant selection are shown in Fig 1. Recruitment was through the Prader-Willi Syndrome Association UK, local clinical services, social care providers supporting people with PWS, and individuals and their families who had previously consented to be contacted about research and known to author AJH.

### Procedure

t-VNS was delivered in accordance with approved protocols for the treatment of epilepsy via a battery-operated stimulator (Cerbomed NEMOS®) connected to an earpiece containing two hemispheric titanium electrodes providing mild electrical stimulation to the cymba conchae of the left ear. In the initial training session, participants and their carers were trained to use the device. Stimulation intensity was increased by 0·1mA steps, from 0·1mA up to a maximum possible 5mA, using a pulse width of 250μs at 25 Hz, until the participant reported a just detectable tingling sensation.[16] t-VNS was worn but switched off during baseline. For the active phase stimulation intensity was adjusted by the participants as previously instructed and worn for four-hours each day. Participants were advised to complete daily stimulation in one four-hour stretch or hourly or two-hourly intervals, and encouraged to incorporate use into their daily routine, but not during sleep. Stimulation history recorded by the device allowed compliance to be verified.

### Measures of behaviour

#### Deviations from the original protocol

Measures of response to challenge (methodology developed previously by author, CO) were initially tried, but are not reported in this paper as no participant found the task challenging enough for it to be effectively completed. The behaviour problems inventory[17], Aberrant Behaviour Checklist[18], and the personal wellbeing index[19] were not completed by carers due to carers having limited time available to them during monthly visits by the researcher.

#### Informant reported temper outbursts

The primary outcome measure was the mean number of temper outbursts per day operationally defined as problem behaviours that escalate to include physical and/or verbal aggression. Carers were asked to record these behaviours in daily behaviour diaries. This method of data collection has been validated by Oliver et al.[20], and can be undertaken in residential or home settings. Support staff and/or family members were already very informed about such behaviours as they had observed them on a regular basis but at the time of recruitment, they were also trained in the definition of outbursts and in using the behaviour diaries. Carers were asked to record daily each time an outburst occurred and to give a detailed description including information on triggers, duration and intensity rating on a 1–5 Likert scale.

Behaviour diaries were used for the duration of the study for three participants (009, 010 and 011). However, due to a high proportion of missing entries early in the study for 003 and 005 (residing in the same setting), detailed daily observation records routinely and mandatorily completed by carers at the residential home were used instead with ethical approval and the participants’ permission. These routinely collected daily observation records detailed the occurrence of problem behaviours, events leading up to, during, and after the behaviour, and the response necessary. For consistency, these records were used throughout baseline and active phases of the study for these two participants and coded using directed qualitative content analysis,[21] with coding categories informed by the daily behaviour diary operationally defined criteria. A 10% sample were rated by two raters. Kappa values of above 0·9 were established for all diary entry criteria for participants 003 & 005, indicating good inter-rater reliability. In the current study, only the occurrence and date of temper outbursts were consistently reported by carer’s, whereas information on triggers, duration, intensity and detailed descriptions of temper outbursts from the daily behaviour diaries were not consistently completed and are therefore not described further.

#### Informant questionnaire

The Challenging Behaviour Interview (CBI)[20] was conducted approximately monthly at participants place of residence as a secondary behavioural outcome measure. The CBI is a structured informant interview that includes questions about the frequency, intensity and impact of behaviours in the previous month and is separated into two sections. Section I documents the occurrence of defined challenging behaviours. Section II records assessments of severity of the behaviours identified in Section I using fourteen questions, each with their own 4-5-point Likert scale (S1 Appendix).

#### Semi-structured interviews

Two semi-structured interviews were conducted with participants and their carers at home: once in the baseline phase and once toward the end of the active phase. Interview schedules (S2–S5 Appendices) comprised key questions about participants’ temper outbursts. These questions were supplemented by prompts from the interviewer, dependent on the interviewees’ responses.

#### Measures of safety

Due to reports of VNS worsening sleep apnoea,[22] sleep was monitored at the Wellcome Trust Clinical Research Facility (CRF) at Addenbrooke’s hospital Cambridge to ensure safety in respect to sleep apnoea using a SomnoscreenTM plus RC system (SOMNOmedics, Germany) twice during each phase.

#### Measure of goal attainment

Goal attainment scaling light[23] (GAS-light) was conducted with carers at home. This measure was added to the original protocol to provide an assessment of t-VNS’s impact beyond that of improved behaviour, specifically, participants’ quality of life and care demands. Baseline scores were retrospectively allocated as -1 to allow for deterioration. Goal attainment was rated at the end of the study. Using Kiresuk & Sherman’s[24] formula, attainment levels were combined to make a single T-score.

### Analysis overview

#### Informant reported temper outbursts

Previous VNS literature suggests a delayed onset of improvements in seizure control when used for treating epilepsy and also in behavioural improvements, ranging from three to twelve months post VNS switch on.[13–15] Therefore, for the purpose of analysis, the active phase for each participant was divided into segments of three months with the aim of establishing if behaviour improves, when it does, and whether all participants improve at a similar time. Participant data was initially pooled into baseline, active phase 1, active phase 2, active phase 3 and active phase 4 groups. A Friedman test was used to determine the significance of group differences in baseline and active phase segments. If a statistical difference was found, a Dunn test with Bonferroni correction evaluated any differences. Since participants lived under different conditions, and whilst all struggled with problem behaviours, the exact characteristics of these behaviours were varied across participants, and for these reasons a single case design was also used with each participant as their own control. Kruskal-Wallis tests were used to evaluate differences in the mean number of outbursts per day at baseline and in each of the three monthly active phase segments for each participant[25]. When a statistical difference was found, a Dunn test with Bonferroni correction was used to assess the significance of these differences.

To evaluate the effect of reducing t-VNS duration by 50%, a Mann-Whitney U test assessed differences between the mean number of outbursts per day in the two-hour t-VNS phase and the last segment of the active phase.

#### Informant questionnaire

A Mann-Whitney U test compared the total CBI scores for Section II during the baseline phase and each three-months of the active phase.

#### Semi-structured interviews

Thematic analysis was carried out following Braun and Clarke.[26] Themes were inductively defined from the raw interview data. Step one involved the researcher familiarizing oneself with the data by re-reading the interview transcripts several times. In step two, open coding was applied to generate initial codes. Similar codes were then collated into categories. In step three, categories and codes were organized into overarching themes which, as per Braun and Clarke,[26] denoted patterned response and/or meaning within the data. Step four involved reviewing, modifying and developing the themes, ensuring all themes were distinct from one another and coherent within the context of the entire dataset. Step five involved defining and naming the themes to communicate succinctly what they concerned. Step six involved producing a report of the analysis, as outlined in the Results.

To demonstrate the robustness and legitimacy of the themes generated, interview extracts are presented in the Results.[26] To ensure validity of results a number of steps were taken: (a) at the time of analysis, the researcher undertaking the analysis (JABW) was experienced in qualitative research, but a novice in PWS and temper outburst research, encouraging the investigation of novel insights; (b) the number of occurrences of each identified theme were totaled to indicate the relative importance of each theme; (c) identified themes were theoretically validated by comparing them with literature on temper outbursts in PWS (see discussion).

## Results

Participant recruitment and retention are shown in Fig 1. Recruitment began on 1^st^ January 2016 and follow-up and study end was on 1^st^ April 2018. Results reported here concern the five participants who completed the study. The demographics, prescribed medications and length of time in each phase for participants are listed in Table 1.

**Table 1:** Participant demographics and medications.

	Sex	Age (yrs.)	Genetic subtype	Duration of BL (months)	Duration of Active (months)	Medication
**003**	M	24.0	delPWS	8	12	Beclometasone nasal, 50mcg, BD Sodium cromoglicate eye drop, 2%, QDS Somatotropine injection, 0.4mg, NOCTE Fexofenadine, 180mcg, OM Risperidone, 1mg, 18:00
**005**	M	23.11	delPWS	10	12	Fluoxetine, 40mg, 08:00 Lamotrigine, 2x 25mg, 08:00 & 18:00 *Lorazepam, 2x 1mg, PRN
**009**	M	41.3	delPWS	5	12	Duolexetine, 30mg, 17:00 Quetiapine, 25mg, PM Risperidone, 2x .125ml, AM & PM
**010**	F	23.8	delPWS	5	12	Keppra, 2x 500mg, AM & PM Metformin, 2g, PM Forxiga, 10mg, PM Atorvastatin, 20mg, daily Gliclazide, 40mg, 2x AM & 1x PM
**011**	F	21.8	delPWS	5	12	Vitamin D, 1000 units, daily Somatotropin, 1.2mg, PM Loestrin 20, x1, daily for 21 days then 7 day break

Age (years. months) at first day of baseline; delPWS, paternal interstitial deletion; BL, baseline phase; month, 30 days; Medication, name, dosage, time administered.

* change in medication at day 84 of 246 baseline phase.

### Compliance

During the active phase, all participants received t-VNS for at least 86% of total possible stimulation time of four hours per day for 360 days [003 (86%); 005 (100%); 009 (93%); 010 (98%); 011 (88%)]. In the two-hour stimulation phase, all participants completed at least 100% of the specified stimulation duration [003 (100%); 005 (108%); 009 (102%); 010 (100%); 011 (105%)].

### Safety

No unexpected events were reported. Mild skin irritation surrounding the electrode was reported in one case, which resolved within two weeks and did not impact usage. No effect on sleep apnoea was observed.

### Efficacy

#### Number of temper outbursts

Initially, mean number of temper outbursts per day were analysed at group level. In SPSS, a Friedman test revealed a significant difference between temper outbursts in the study phases, *χ*^2^ (4) = 14.10, *p* = .007. Dunn-Bonferroni post hoc tests were carried out and there were significant differences between baseline and active phase four (*p* = .014, d = 1.01) and active phase one and active phase four (*p* = .014, d = 1.01) after Bonferroni adjustments.

**Fig 2:**
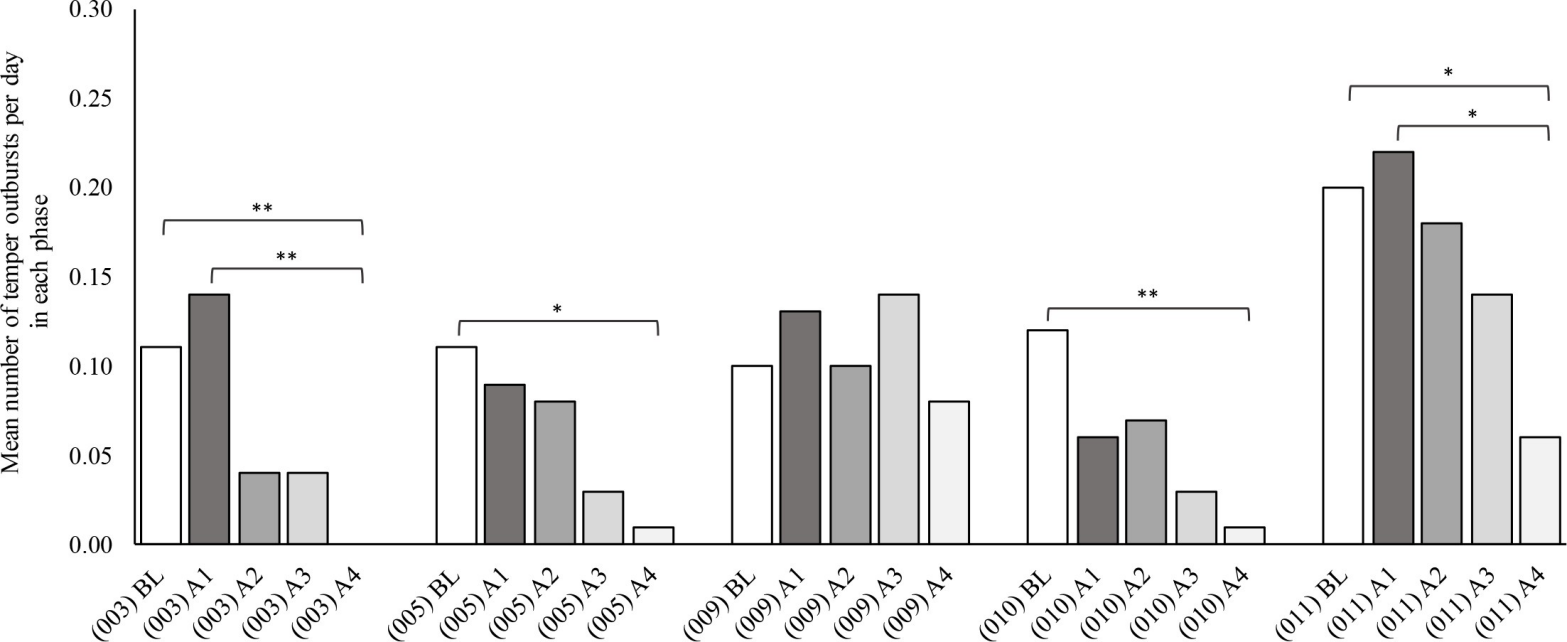
Mean number of temper outbursts per day in each phase for each participant. BL, baseline phase; A1, first three months of active phase; A2, second three months of active phase; A3, third three months of active phase; A4, fourth three months of active phase. Pairwise comparisons Dunn test. BL compared to A4: 003 (p = ·009); 005 (p = ·021); 010 (p = ·009); 011 (p = ·045). A1 compared to A4: 003 (p = ·003); 011 (p = ·034).

Fig 2 shows the mean number of temper outbursts per day in the baseline and active phases for each participant. Mean number of temper outbursts per day were then analysed for each individual participant. In SPSS, using the Kruskal-Wallis test a statistically significant difference was found between baseline and active phase four for 003, 005, 010 and 011 [003 (*p =* <·001, d = ·18); 005 (*p =* ·013, d = ·16); 010 (*p =* ·007, d = ·21); 011 (*p =* ·028, d = ·18)]. Participant 009 showed no significant difference in temper outbursts between the baseline and active phases (*p =* ·679).

#### Informant questionnaires

Behaviours present in section I and the mean total CBI scores for section II during the baseline and active phase four for each participant are shown in Table 2. In SPSS, a Mann-Whitney U tests revealed a statistically significant reduction in CBI scores from baseline to active phase four for 003, 005, 010 and 011. [003 (*p* = ·02); 005 (*p* = ·046); 010 (*p* = ·022); 011 (*p* = ·028)]. No significant change was observed for 009 (*p* = ·471).

**Table 2:** Types of behaviour’s present and mean total CBI scores for each participant in baseline and active phase 4 as reported in the CBI, and percentage of days t-VNS was worn by each participant.

	Behaviours Present		Mean total CBI scores per phase		
	BL	A4	BL	A4	*P* ^a^
**003**	Verbal aggression; Physical aggression; Destruction of property; Self injury; Stereotyped behaviours; Stealing	Verbal aggression	31.67 (15.07)	2.67 (4.62)	0.02
**005**	Verbal aggression; Physical aggression; Destruction of property; Self injury; Stereotyped behaviours;	Verbal aggression	24.67 (5.51)	1.67 (2.89)	0.046
**009**	Verbal aggression	Verbal aggression	11 (1.83)	15.67 (8.14)	0.471
**010**	Verbal aggression		15.6 (2.97)	0 (0)	0.022
**011**	Verbal aggression; Physical aggression; Stealing		19 (5.1)	0 (0)	0.028

BL, baseline; A4, active phase 4; Active, entire active phase of 4-hours daily stimulation

^a^ P-value of Mann-Whitney U test comparing total CBI scores between baseline and active phase 4 for each participant.

#### Observations of participant behaviour:

Thematic analysis identified ten sub-themes from the semi-structured interviews with carers and participants (Table 3). These sub-themes were organised into three overarching themes; Control of emotions; Interactions with environment/setting; and Regulation of behaviour (S6 Appendix).

**Table 3:** Selected examples of verbatim quotes for each identified theme.

Study Phase	Sub-theme	Verbatim Quotes
**Baseline**	Uncontrolled mood	"He is very, very aggressive, extremely anxious, and it’ll be over something very, very small, maybe he couldn’t find his telephone" [VNS005] "..there’s no rhyme or reason for her mood sometimes . . .we only need to say one careless comment . . .and it will escalate suddenly" [VNS010]
	Rigidity	"As long as he is aware of what he’s doing… he’s fine, if there’s a change…that can really upset him, he can become quite agitated and anxious" [VNS003] "She likes to have total control and when she feels that what we are saying or doing is taking away even a fraction of that control that is enough to send her into meltdown" [VNS010]
	Necessity for planning	"…So as long as he’s prepped before, if we can, then it does limit [temper outbursts]" [VNS003] "She only becomes angry when things don’t go as planned" [VNS011]
	Behaviour impacts everyday life	"Does his behaviour affect his own plans and activities at all, does it disrupt them of delay them?"—"It can delay them" [VNS003] "We don’t like doing it [taking participant out for meals] because that is always a distinct possibility of having a flashpoint [of behaviour] there because of the choice of what’s on the menu" [VNS010]
	No opportunity for intervention	"She shuts down. I don’t know if she can actually process what you’re saying to her" [VNS010] " . . .there’s just no reasoning with her…" [VNS011]
**Active**	Reduced outbursts	"…so before . . .it [a temper outburst] could go on for like two, three, four plus hours, and now it’s literally like quick outburst …and then he sort of stops and, and sort of thinks a little bit" [VNS005] "in the last five months or four months . . .it [a temper outburst] seems to have been far less often..they’re lasting fewer minutes than they used to I think" [VNS010]
	Controlled mood	"…before it could be the slightest little thing…y’know scream shout . . .now if you say to him “[3] it’s just gonna be five minutes y’know we’re almost there”, he goes 'OK I’ll wait‴ [VNS003] " . . .she came over to me and we just talked about it and I explained it in a different way and she calmed…she just seemed to accept it which a few weeks ago I don’t think she would have" [VNS011]
	Flexibility	" . . .any changes, anything slight would trigger his anxiety’s off, umm since he’s been on the VN we’ve seen a massive change in [participant]. He’s a little bit more tolerant with timing" [VNS003] "she’s accepting more changes now than she used to I think…without a fuss" [VNS010]
	Behaviour positively impacts everyday life	"If he’s too heightened then obviously it may deem unsafe for him to go…we haven’t had to do something like that with [participant] for the last six months" [VNS003] "I think she’s happier in herself . . . she’s not falling out with [friend] as much as she used to" [VNS010]
	Opportunity for intervention	" . . .before you couldn’t challenge him on certain things, where you needed to, but now you can and he’s prepared to sit and listen" [VNS003] "I think we’ve got more breathing space because she often doesn’t react immediately…it can give you time to think . . .of some way of distracting her or diffusing the situation" [VNS010]

In the active compared to baseline phase, informants reported an increased ability to regulate emotions described in terms of reduced rash emotional responses and behavioural volatility. This improved emotional control was described as facilitating regulation of behaviour and resulting in an increased ability to process a situation and listen to advice, allowing the person with PWS to benefit from interventions (verbal prompts etc.). As a consequence of these reported improvements, participants’ abilities to manage and respond well to conditions that would have previously triggered a temper outburst improved, resulting in less frequent and lengthy temper outbursts.

### Stimulation parameters

#### Temper outbursts during reduced t-VNS duration

**Fig 3:**
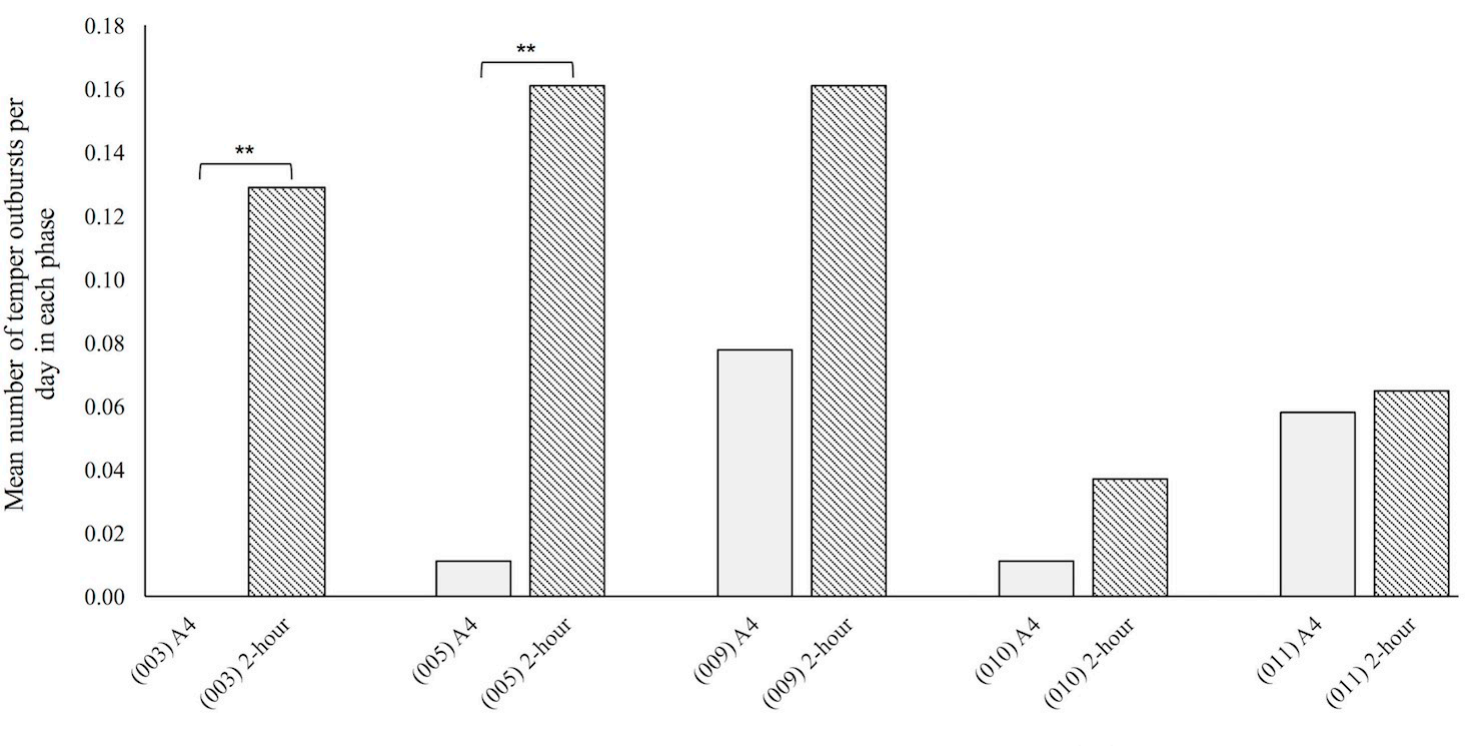
Mean number of temper outbursts per day during active phase 4 and 2-hour stimulation phase. A4, last three months of active phase; 2-hour, t-VNS worn for 2-hour in this stimulation phase. P-values of Mann-Whitney U test: 003 (p = ·000); 005 (p = ·001).

All four participants showing improvement reduced their use of t-VNS from four to two hours per day after 12 months of active stimulation. Although 009 had not shown a significant improvement in behaviour some changes had been described and for this reason his daily use of VNS was initially reduced rather than just stopped. A Mann-Whitney U test revealed that in the two-hour t-VNS phase 003 and 005 displayed significantly more temper outbursts than in active phase four (Fig 3). No significant difference was found for 009, 010 or 011 (Fig 3). However, carers reported 010 and 011 as more irritable and unable to be reasoned with during the reduced t-VNS phase compared to active phase four. For 009 a numerical increase in temper outbursts was reported in behaviour diaries and anecdotally by carers during this reduction phase. After one month on two-hour stimulation all participants asked to increase the stimulation back to four-hours per day. In line with what had been approved by the ethics committee all had their stimulation time increased and all four who improved chose to continue on the t-VNS after the study ended and were referred to their General Practitioner for subsequent monitoring.

#### Goal attainment scores

Table 4 shows the GAS-light T-scores for each participant. There was a numerical increase in T-scores for participants 003, 005, 010 and 011. There was no change in T-scores for 009.

**Table 4:** GAS light values for each participant in baseline and active phase 4.

	GAS light values							
	Goal 1^a^		Goal 2^b^		Goal 3^c^		T-score	
	BL	A4	BL	A4	BL	A4	BL	A4
**003**	-1	2	-1	2	-1	1	36.3	68.3
**005**	-1	2	-1	2	-1	1	36.3	68.3
**009**	-1	-1	-1	-1	-1	-1	36.3	36.3
**010**	-1	2	-1	2	-1	1	36.3	72.8
**011**	-1	1	-1	1	-1	1	36.3	63.7

BL, baseline; A4, active phase 4. Attainment for each goal was rated where: 0, expected level of achievement; +1, a little better than expected; +2, a lot better than expected; -1, no change; -2, worsening. T-score derived from Kiresuk & Sherman’s formula.[24]

^a^ Goal 1, reduce number of temper outbursts for the participant

^b^ Goal 2, improve the participants quality of life

^c^ Goal 3, reduce the participants care demands.

## Discussion

This is the first study to systematically investigate the use of t-VNS to ameliorate the temper outbursts and related behaviours in PWS. Participants received VNS via an externally worn device, rather than a surgically implanted device as has previously been reported[10], and behaviour was recorded both contemporaneously in a naturalistic environment and in retrospect using informant questionnaires and interviews. Our results show that t-VNS was associated with significant reductions in temper outbursts in four out of five participants, underpinned by improvements in emotional control, improved responses to interventions and increased ability to effectively manage previously outburst-stimulating situations. Reduced severity and impact of challenging behaviour was also demonstrated in these four participants. Temper outbursts in PWS begin in childhood and continue into adult life[2], having a severe effect on quality of life of people with PWS and their carers. This level of reduction in occurrence and severity of these behaviours is significant. These findings concur with previous reports suggesting that VNS reduces maladaptive behaviour in PWS[10] and other neurodevelopmental disorders.[13–15] No new side effects were reported, suggesting that the safety profile in PWS is similar to VNS for epilepsy. Moreover, improvements in GAS-light scores indicate reduced care demands and improved quality of life for participants, with carer expectations of the intervention being exceeded for all participants who improved.

Initial interviews revealed that a key trigger for temper outbursts in all participants was change, as is reported in previous literature.[3,7–9] All participants who displayed fewer temper outbursts after t-VNS reported improvements in behavioural flexibility and ability to accept change. Increased responsiveness to interventions aimed at placating participants prior to a potential temper outburst was also reported.

The observation of statistically significant behavioural improvements occurring by approximately month nine of the active phase indicates a mode of action that requires time to develop, in line with behavioural improvements observed in people with ASD and epilepsy.[13–15] However, lower compliance for 003, 010 and 011 early in the active phase may have contributed to later onset of improvement. This finding, combined with the observation that a 50% reduction in stimulation time resulted in an increase in outbursts, indicates that continued stimulation at the initial level is required to maintain a therapeutic effect.

It is unclear why 009 did not show behavioural improvement. However, at 41 years, 009 was older than other participants. Research on behaviour in older adults with PWS over the age of 30 is scarce but does indicate a changing profile with reduced outburst-related behaviours with age.[27] A specific functional reinforcement of outbursts for 009 may also be implicated, with desired one-to-one care only accessed when necessitated by such behaviours. The behaviours displayed by 009 were no worse than others but it was of note that he had been prescribed the highest doses of antipsychotic medications compared to all other participants.

### Limitations and challenges

There were several significant challenges in the design and undertaking of the study. First, a case series modified ABA repeated measures design was chosen, rather than a double-blind trial with sham and active stimulation phases. This was for several reasons: the rarity of PWS and consequent likelihood of small sample size, the need to undertake the study in a naturalistic setting resulting in the possibility for major differences between individual circumstances, and uncertainty about the length of time in the active stimulation phase required before effects might be seen, and the non-blind nature of the study imposed by the stimulus protocol requiring stimulation to be detectable. The lack of a sham stimulation arm means that the possibility that these marked improvements were a consequence of a placebo effect and/or because of observer bias cannot be ruled out. However, significant improvements were only apparent after nine months of stimulation for all four improved participants. In addition, although carers were aware improvements may be delayed, similar patterns of improvement were reported across three separate care settings (two participants resided together). In addition, these observed improvements were informally reported to have continued months after the study ended. These factors, together with homogeneous effect sizes, suggest improvements in behaviour are unlikely to be due to a placebo effect or a consequence of observer bias.

Secondly, we were dependent upon support staff for data collection using the daily behaviour diary. This was in addition to their normal data recording. Early after recruitment of two participants it was apparent that the diaries were not being completed and we obtained ethical approval and consent of the participants to use the daily records that staff routinely kept. This same method of contemporaneous data collection (existing behavioural records) was used in both phases for each participant ensuring consistency across baseline and active phases. Additionally, inter-rater reliability for this form of data collection in these two participants was high.

Thirdly, a key inclusion criterion was that participants had temper outbursts. Compliance was good for participants who completed the study (>86%), with all participants opting to continue t-VNS post study end. However, inability to comply had a large impact on dropout rates early in the baseline phase. Placement breakdown and/or change in support circumstance was the main cause of drop out, suggesting that a facilitative care environment may be crucial for t-VNS acceptability. It was partly for this reason that the length of baseline phases varied between participants and also the active phase was extended to one year, ensuring improvements were unlikely to be transient responses to changing circumstances. Indeed, a medication change during baseline for 005 may have contributed to reduced outbursts in active phase one; however, this reduction was minor compared to the difference observed between baseline and active phase four. This extension also ensured that for 009, insufficient stimulation duration was unlikely to be responsible for lack of improvement.

Finally, stimulation intensity in this study adhered to recommended t-VNS parameters.^16^ However, it is possible that different frequencies, pulse widths or amplitudes may more optimally stimulate the vagus nerve.[28] It is important to note that t-VNS is licensed for the treatment of depression and, although no participant met criteria for co-morbid mood disorder, a moderating effect on mood may be implicated.

### Future research

For t-VNS to become fully accepted as a treatment for behaviour problems in people with PWS further studies are required. First, this study did not contain a sham condition. Future work should if possible include a sham condition to account for any potential placebo effects or observer bias that could have been present in this study. Secondly, this is a small study and replication is needed with an extension of the age-range of the participants downwards to include children with PWS. Thirdly, further work is needed to refine the electrode design and to develop strategies that maximises compliance in this group who have a history of problem behaviours. Fourthly, additional data on the wider benefits of treatment on quality of life, beneficial effects on family and/or paid carers, and savings on health and care costs are needed in order to further build the case for making this treatment routinely available and funded through the health insurance systems or national health services. Fifthly, in this study we opted to use the stimulus protocol recommended for treating epilepsy but this may not be the optimum for effective neuromodulation. Finally, an understanding of the mechanism of action is required both to inform the stimulus protocol and also to determine whether this treatment might be extended to other clinical groups with similar or related problem behaviours.

From the perspective of better understanding why problem behaviours are common among people with PWS and also in those with other neurodevelopmental disorders this observation of the beneficial effects of t-VNS indicates a need to move away from seeing them as primarily shaped and re-inforced by experience but rather they are the result of impaired emotional regulation that we suggest is a consequence of autonomic nervous system dysfunction and the impact of such dysfunction centrally in the brain. The polyvagal theory[29] proposes that a brainstem centre regulating the vagal nerve, often labelled the ventral vagal complex, acts as the ‘command centre’ for the vagal pathways mediating a calm behavioural state. Participants’ increase in ability to maintain calmness post t-VNS suggests that stimulating the vagal nerve’s afferent pathways may partially ameliorate the function of potentially perturbed vagal pathways.

Woodcock et al. have proposed that change to routines or expectations place a higher than normal cognitive load on individuals with PWS, due to a proposed deficit in attention shifting ability, and it is the excess cognitive demand that results in loss of control.[3,7–9] Such rigidity in thinking whilst dysfunctional may be a natural and adaptive response to feeling under threat (e.g. in anxiety threat vigilance is increased[30] and induced anxiety is associated with cognitive rigidity).[31] Consequently, what may seem to be a minor threat stimulus (a change in environment) may trigger a full fight response in the form of an outburst, and is in line with anecdotal reports[32] of the behaviour of monitoring fairness by people with PWS in resource allocation in group living settings. One model would be that t-VNS produces a moderating effect on emotional regulation, potentially by affecting the response to the high cognitive load experienced by people with PWS under certain circumstances.

Increased vagal tone, and thus increase in parasympathetic activation may also be associated with changes in neurotransmitter systems, noradrenaline, acetylcholine (Ach), and gamma-aminobutyric acid (GABA).[33] Of particular significance, tVNS has been associated with increases in GABA being observed in patients with epilepsy after nine months of stimulation[34] as well as elevated GABAergic motor cortical activity. [35] Rice et al. have also shown that brain GABA levels were reduced in people with PWS who had emotional and behavioural problems, including temper outbursts.[36] This evidence, coupled with this study’s findings that t-VNS reduces such behaviours, suggests that the GABAergic system (in association with vagal tone) may play an important role in mediating temper outbursts. Further studies are required to explore these various explanations.

## Conclusions

This study has demonstrated that VNS delivered by an external stimulus for four hours each day has a significant therapeutic effect on temper outbursts in people with PWS that extends to improvements in life more generally. Further analysis and investigation of the mechanisms that underpin t-VNS’s efficacy on temper outbursts in PWS are required as are investigations into the optimum dose of t-VNS given the length of time before improvement and the behavioural deterioration observed when the four-hour dose was halved. Future research should also address the lack of a sham condition in this current study. All four people with PWS who improved have continued treatment and the effect on their lives has been reported as substantial.

## Supporting information

S1 Appendix Challenging behaviour interview (CBI) questions and rating scales. (DOCX)

S2 Appendix Active phase interview questions for participant. (DOCX)

S3 Appendix Active phase interview questions for parent/support worker. (DOCX)

S4 Appendix Baseline interview questions for participant. (DOCX)

S5 Appendix Baseline interview questions for parent/support worker. (DOCX)

S6 Appendix Thematic analysis of semi-structured interviews exploring reported behavioural changes for participants 003, 005, 010, & 011. (DOCX)

S7 Appendix TREND checklist. (PDF)

S8 Appendix Study protocol. (DOCX)

S9 Appendix Number of temper outbursts per day for each participant. (XLSX)

S10 Appendix CBI scores in the baseline phase and active phase four for each participant. (XLSX)

## References

[1] Whittington J E., Holland A, J., Webb T., Butler J., Clarke D. & Boer H. Population prevalence and estimated birth incidence and mortality rate for people with Prader-Willi syndrome in one UK Health Region. *J*. *Med*. *Genet*. 38, 792–798 (2001). 10.1136/jmg.38.11.792 11732491 PMC1734766

[2] Holland A. J., Whittington J, E., Butler J., Webb T., Boer H. & Clarke D. Behavioural phenotypes associated with specific genetic disorders: evidence from a population-based study of people with Prader-Willi syndrome. *Psychol. Med*. 33, 141–153 (2003). 10.1017/s0033291702006736 12537045

[3] Woodcock K., Oliver C. & Humphreys G. Associations between repetitive questioning, resistance to change, temper outbursts and anxiety in Prader-Willi and Fragile-X syndromes. *J*. *Intellect*. *Disabil*. *Res*. 53, 265–278 (2009). 10.1111/j.1365-2788.2008.01122.x 18771510

[4] Mazaheri M. M., Rae-Seebach R. D., Preston H. E., Schmidt M., Kountz-Edwards S., Field N. et al The impact of Prader-Willi syndrome on the family’s quality of life and caregiving, and the unaffected siblings’ psychosocial adjustment. *J*. *Intellect*. *Disabil*. *Res*. 57, 861–873 (2013). 10.1111/j.1365-2788.2012.01634.x 23057501

[5] Bittel D. C. & Butler M. G. Prader–Willi syndrome: clinical genetics, cytogenetics and molecular biology. *Expert Rev Mol Med*. 7, 1–20 (2005). 10.1017/S1462399405009531 PMC6750281 16038620

[6] Cassidy S. B., Forsythe M., Heeger S., Nicholls R. D., Schork N., Benn P. et al Comparison of phenotype between patients with Prader-Willi syndrome due to deletion 15q and uniparental disomy 15. *Am*. *J*. *Med*. *Genet*. 68, 433–440 (1997). 9021017

[7] Woodcock K. A., Oliver C. & Humphreys G. Task-switching deficits and repetitive behaviour in genetic neurodevelopmental disorders: Data from children with Prader—Willi syndrome chromosome 15 q11—q13 deletion and boys with Fragile X syndrome. *Cogn. Neuropsychol*. 26, 172–194 (2009). 10.1080/02643290802685921 19221920

[8] Woodcock K. A., Oliver C. & Humphreys G. W. The relationship between specific cognitive impairment and behaviour in Prader-Willi syndrome. *J*. *Intellect*. *Disabil*. *Res*. 55, 152–171 (2011). 10.1111/j.1365-2788.2010.01368.x 21199046

[9] Woodcock K. A., Humphreys G. W., Oliver C. & Hansen P. C. Neural correlates of task switching in paternal 15q11-q13 deletion Prader-Willi syndrome. *Brain Res*. 1363, 128–142 (2010). 10.1016/j.brainres.2010.09.093 20920489

[10] Manning K. E., McAllister C. J., Ring H. A., Finer N., Kelly C. L., Sylvester K. P. et al Novel insights into maladaptive behaviours in Prader-Willi syndrome: Serendipitous findings from an open trial of vagus nerve stimulation. *J*. *Intellect*. *Disabil*. *Res*. 60, 149–155 (2016). 10.1111/jir.12203 26018613 PMC4950305

[11] Galli R., Bonanni E., Pizzanelli C., Maestri M., Lutzemberger L., Giorgi F. S. et al Daytime vigilance and quality of life in epileptic patients treated with vagus nerve stimulation. *Epilepsy Behav*. 4, 185–191 (2003). 10.1016/s1525-5050(03)00003-9 12697145

[12] Kossoff E. H. & Pyzik P. L. Improvement in alertness and behavior in children treated with combination topiramate and vagus nerve stimulation. *Epilepsy Behav*. 5, 256–259 (2004). 10.1016/j.yebeh.2003.12.008 15123029

[13] Warwick T. C., Griffith J., Reyes B., Legesse B. & Evans M. Effects of vagus nerve stimulation in a patient with temporal lobe epilepsy and Asperger syndrome: Case report and review of the literature. *Epilepsy Behav*. 10, 344–347 (2007). 10.1016/j.yebeh.2007.01.001 17300990

[14] Hull M. M., Madhavan D. & Zaroff C. M. Autistic spectrum disorder, epilepsy, and vagus nerve stimulation. *Child’s Nerv*. *Syst*. 31, 1377–1385 (2015). 25922052 10.1007/s00381-015-2720-8

[15] Murphy J. V., Wheless J. W. & Schmoll C. M. Left vagal nerve stimulation in six patients with hypothalamic hamartomas. *Pediatr*. *Neurol*. 23, 167–168 (2000). 10.1016/s0887-8994(00)00170-3 11020644

[16] Ellrich J. Transcutaneous vagus nerve stimulation. *Eur*. *Neurol*. *Rev*. 6, 254–256 (2011).

[17] Rojahn J., Matson J. L., Lott., Esbensen A. J. & Smalls Y. The Behavior Problems Inventory: an instrument for the assessment of self-injury, sterotyped behavior, and aggression/destruction in individuals with developmental disabilities. *J Autism Dev Disord*. 31, 577–88 (2001). 10.1023/a:1013299028321 11814269

[18] Aman M. G., Singh N. N., Stewart A. W. & Field C. J. The aberrant behavior checklist: A behavior rating scale for the assessment of treatment effects. *Am*. *J*. *Ment*. *Defic*. 89, 485–491 (1985). 3993694

[19] Cummins R. A. & Lau A. D. L. Personal wellbeing index—Intellectual Disability. 3rd Edition Victoria, Australia: School of Psychology, Deakin University (2005).

[20] Oliver C., McClintock K., Hall S., Smith M., Dagnan D. & Stenfert-Kroese B. Assessing the Severity of Challenging Behaviour: Psychometric Properties of the Challenging Behaviour Interview. *J*. *Appl*. *Res*. *Intellect*. *Disabil*. 16, 53–61 (2003).

[21] Hsieh H-F. & Shannon S, E. Three approaches to Qualitative Content Analysis. *Qual*. *Health Res*. 15, 1277–1288 (2005). 10.1177/1049732305276687 16204405

[22] Murray B. J., Matheson J. K. & Scammell T. E. Effects of vagus nerve stimulation on respiration during sleep. *Neurology* 57, 1523–1524 (2001). 10.1212/wnl.57.8.1523 11673612

[23] Turner-Stokes L. Goal Attainment Scaling (GAS) in Rehabilitation: A practical guide Clinical Rehabilitation. *Clin. Rehabil*. 23, 362–270 (2009). 10.1177/0269215508101742 19179355

[24] Kiresuk T, J. & Sherman R, E. Goal attainment scaling: A general method for evaluating comprehensive community mental health programs. *Community Ment*. *Health J*. 4, 443–453 (1968). 10.1007/BF01530764 24185570

[25] Dugard P., File P., & Todman J. Single-case and small-n experimental designs: a practical guide to randomization tests. Second edition Routledge: Hove (2001)

[26] Braun V. & Clarke V. Using thematic analysis in psychology Using thematic analysis in psychology. *Qual*. *Res*. *Psychol*. 3, 77–101 (2008).

[27] Dykens E, M., Hodapp R, M., Walsh K. & Nash L, J. Profiles, Correlates, and Trajectories of Intelligence in Prader-Willi Syndrome. *J*. *Am*. *Acad*. *Child Adolesc*. *Psychiatry* 31, 1125–1130 (1992). 10.1097/00004583-199211000-00022 1429416

[28] Usami K., Kawai K., Sonoo M. & Saito N. Scalp-recorded evoked potentials as a marker for afferent nerve impulse in clinical vagus nerve stimulation. *Brain Stimul*. 6, 615–623 (2013). 10.1016/j.brs.2012.09.007 23088852

[29] Porges S.W. & Furman S. A. The Early Development of the Autonomic Nervous System Provides a Neural Platform for Social Behavior: A Polyvagal Perspective. *Infant Child Dev*. 20, 106–118 (2011). 10.1002/icd.688 21516219 PMC3079208

[30] Bar-Haim Y., Lamy D., Pergamin L., Bakermans-Kranenburg M. J. & Van Ijzendoorn M. H. Threat-related attentional bias in anxious and nonanxious individuals: A meta-analytic study. *Psychol. Bull*. 133, 1–24 (2007). 10.1037/0033-2909.133.1.1 17201568

[31] Robinson O. J., Letkiewicz A. M., Overstreet C., Ernst M. & Grillon C. The effect of induced anxiety on cognition: threat of shock enhances aversive processing in healthy individuals. *Cogn Affect Bheav Neurosci*. 11, 217–227 (2011). 10.3758/s13415-011-0030-5 PMC3169349 21484411

[32] Prader-Willi California Foundation. Prader-Willi Syndrome: Overview of Food & Behaviour Management.

[33] Van Leusden J. W. R., Sellaro R. & Colzato L. S. Transcutaneous Vagal Nerve Stimulation (tVNS ): a new neuromodulation tool in healthy humans? *Front. Psychol*. 6, (2015). 10.3389/fpsyg.2015.00102 PMC4322601 25713547

[34] Ben-Menachem E., Hamberger A., Hedner T., Hammond E. J., Uthman B. M., Slater J. et al Effects of vagus nerve stimulation on amino acids and other metabolites in the CSF of patients with partial seizures. *Epilepsy Res*. 20, 221–227 (1995). 10.1016/0920-1211(94)00083-9 7796794

[35] Capone F., Assenza G., Di Pino G., Musumeci G., Ranieri F., Florio L. et al The effect of transcutaneous vagus nerve stimulation on cortical excitability. *J*. *Neural Transm*. 122, 679–685 (2015). 10.1007/s00702-014-1299-7 25182412

[36] Rice L. J., Lagopoulos J., Brammer M. & Einfeld S. L. Reduced Gamma-Aminobutyric Acid Is Associated With Emotional and Behavioral Problems in Prader–Willi Syndrome. *Am J Med Genet B Neuropsychiatr Genet*. 171, 1041–1048 (2016). 10.1002/ajmg.b.32472 27338833

