# Supplementary material for: Transcutaneous vagus nerve stimulation (t-VNS): A novel effective treatment for temper outbursts in adults with Prader-Willi Syndrome indicated by results from a non-blind study
Source: PLoS One. 2019 Dec 3;14(12):e0223750. doi: 10.1371/journal.pone.0223750 (PMC6890246; doi:10.1371/journal.pone.0223750)
Supplement: S1 Appendix — (DOCX) [file pone.0223750.s001.docx]

**S1 Appendix.** **Challenging behaviour interview (CBI) questions and rating scales.**
