# Supplementary material for: Transcutaneous vagus nerve stimulation (t-VNS): A novel effective treatment for temper outbursts in adults with Prader-Willi Syndrome indicated by results from a non-blind study
Source: PLoS One. 2019 Dec 3;14(12):e0223750. doi: 10.1371/journal.pone.0223750 (PMC6890246; doi:10.1371/journal.pone.0223750)
Supplement: S2 Appendix — (DOCX) [file pone.0223750.s002.docx]

**S2 Appendix. Active phase interview questions for participant.**

Participant ID:

Date:

Try to get examples of times where differences are if relevant and possible.

1. What would you say your mood and behaviour are like?
2. Do you think you struggle with your mood and behaviour?
3. In what way?
4. Do you think your mood or behaviour has been different since we switched on the VNS? If yes, in what way?
5. Do you think that anything else has changed since we switched on the VNS?
6. What sort of things make you upset or frustrated? What sort of things make it hard for you to control your behaviour?
7. Are there any particular places where you find it very difficult to control your mood or behaviour? Or particular things that often make you get angry or upset?
8. When you are finding it a bit difficult to control your behaviour or are getting upset, what happens? What do you do?
9. How often does this happen?
10. What are the first things you feel when you are starting to get upset or frustrated or lose control of your behaviour?
11. Is there anything that can help you calm down?
12. What about times when this doesn’t work? Are there ever times when you just can’t find a way to calm down?
13. What happens then?
14. How often does this happen?
15. Does the way you struggle sometimes to control your behaviour get in the way of you doing things or getting on with other people? Can you tell me about that?
16. Do you think your life would be different if you didn’t struggle with your mood or behaviour or [particular situations they have stated] like this? How do you think it would be different?
