# Supplementary material for: Transcutaneous vagus nerve stimulation (t-VNS): A novel effective treatment for temper outbursts in adults with Prader-Willi Syndrome indicated by results from a non-blind study
Source: PLoS One. 2019 Dec 3;14(12):e0223750. doi: 10.1371/journal.pone.0223750 (PMC6890246; doi:10.1371/journal.pone.0223750)
Supplement: S3 Appendix — (DOCX) [file pone.0223750.s003.docx]

**S3 Appendix. Active phase interview questions for parent/support worker.**

Participant ID:

Relationship to participant:

How long have they been caring for X:

Date:

TRY TO GET SPECIFIC EXAMPLES OF BEFORE AND NOW OR TIMES WHEN CARER NOTICED CHANGE IN BEHAVIOUR ETC.

1. Tell me a bit about what X is like day to day? What is his/her ‘state of mind’ or mood like? Has this been any different since the VNS was switched on?
2. Do you think there have been changes in X’s mood and behaviour since we switched on the VNS?
3. Any other changes since switching on the VNS? (e.g. weight)
4. What presents the biggest problems for X? Has this changed at all?
5. What things make it most difficult to support X?
6. What kind of behavioural struggles does X have? What distresses him/her? Is this any different to before the VNS was turned on?
7. What sort of things usually trigger a behaviour for X? Has this changed at all?
8. Are there places, events or tasks that you have to or try to avoid with X? Has this changed at all?
9. When X has a behaviour, what happens? Is this any different?
10. How often does this happen? Is this more, less or the same since the VNS was switched on?
11. How can you tell if a behaviour is about to happen?
12. What things can you do at this point to try to resolve it or stop it getting out of hand? Has what you need to do or how often you need to do this changed at all?
13. What about lower level behaviours? Does X show difficult behaviours that don’t always lead to a full outburst but cause problems for him/her? Again, any changes with VNS?
14. How often do these happen?
15. How do these behaviours affect the people around X or X’s own plans or activities? Any change?
16. How would X’s life be different if he/she didn’t struggle with his/her mood or behaviour like this? OR how has it changed?
