# Supplementary material for: Transcutaneous vagus nerve stimulation (t-VNS): A novel effective treatment for temper outbursts in adults with Prader-Willi Syndrome indicated by results from a non-blind study
Source: PLoS One. 2019 Dec 3;14(12):e0223750. doi: 10.1371/journal.pone.0223750 (PMC6890246; doi:10.1371/journal.pone.0223750)
Supplement: S5 Appendix — (DOCX) [file pone.0223750.s005.docx]

**S5 Appendix. Baseline interview questions for parent/support worker.**

Participant ID:

Relationship to participant:

How long have they been caring for X:

Date:

1. Tell me a bit about what X is like day to day? What is his/her ‘state of mind’ or mood like?
2. What presents the biggest problems for X?
3. What things make it most difficult to support X?
4. What kind of behavioural struggles does X have? What distresses him/her?
5. What sort of things usually trigger a behaviour for X?
6. Are there places, events or tasks that you have to or try to avoid with X?
7. When X has a behaviour, what happens?
8. How often does this happen?
9. How can you tell if a behaviour is about to happen?
10. What things can you do at this point to try to resolve it or stop it getting out of hand?
11. What about lower level behaviours? Does X show difficult behaviours that don’t always lead to a full outburst but cause problems for him/her?
12. How often do these happen?
13. How do these behaviours affect the people around X or X’s own plans or activities?
14. How would X’s life be different if he/she didn’t struggle with his/her mood or behaviour like this?
