# Supplementary material for: Transcutaneous vagus nerve stimulation (t-VNS): A novel effective treatment for temper outbursts in adults with Prader-Willi Syndrome indicated by results from a non-blind study
Source: PLoS One. 2019 Dec 3;14(12):e0223750. doi: 10.1371/journal.pone.0223750 (PMC6890246; doi:10.1371/journal.pone.0223750)
Supplement: S6 Appendix — (DOCX) [file pone.0223750.s006.docx]

**S6 Appendix. Thematic analysis of semi-structured interviews exploring reported behavioural changes for participants 003, 005, 010, & 011.**

|  | **Overarching theme** | **Sub-theme** | **Code** |
| --- | --- | --- | --- |
| **Baseline phase** | Control of emotions | Uncontrolled mood | minor triggers |
|  |  |  | volatile behaviour |
|  |  |  | disproportionate responses |
|  |  |  | continual food-related anxiety |
|  |  |  | withdrawn |
|  |  |  | repetitive questioning |
|  |  |  | verbal aggression |
|  |  |  | jumping to conclusions |
|  | Interactions with environment/setting | Rigidity | need for routine |
|  |  |  | change causes distress |
|  |  |  | stubbornness |
|  |  | Necessity for planning | no outburst if a plan is adhered to |
|  |  |  | need for prior warning of changes |
|  |  | Behaviour impacts everyday life | behaviour delays plans |
|  |  |  | avoidance of certain places |
|  | Regulation of behaviour | No opportunity for intervention | interventions rarely work |
|  |  |  | can't be challenged |
|  |  |  | unable to be negotiated with |
|  |  |  | intervening can make behaviour worse |
| **Active phase** | Control of emotions | Reduced outbursts | lower frequency of outbursts |
|  |  |  | brief outbursts |
|  |  | Controlled mood | no physical aggression |
|  |  |  | little to no verbal aggression |
|  |  |  | no disproportionate responses |
|  |  |  | accepts previous triggers |
|  |  |  | listens before jumping to conclusions |
|  |  |  | can accept being denied food |
|  |  |  | can control behaviour |
|  |  |  | general calm mood |
|  |  |  | reduced anxiety |
|  |  |  | able to calm down |
|  | Interactions with environment/setting | Flexibility | can accept change |
|  |  |  | no preparation/notice needed |
|  |  | Behaviour positively impacts everyday life | behaviour does not affect plans |
|  |  |  | not falling out with friends |
|  |  |  | no food seeking or worrying about food |
|  | Regulation of behaviour | Opportunity for intervention | can be challenged |
|  |  |  | can understand/process the situation |
|  |  |  | outburst can be prevented at first signs |
|  |  |  | can listen to advice |
|  |  |  | able to be communicated with |
