# Supplementary material for: Transcutaneous vagus nerve stimulation (t-VNS): A novel effective treatment for temper outbursts in adults with Prader-Willi Syndrome indicated by results from a non-blind study
Source: PLoS One. 2019 Dec 3;14(12):e0223750. doi: 10.1371/journal.pone.0223750 (PMC6890246; doi:10.1371/journal.pone.0223750)
Supplement: S8 Appendix — (DOCX) [file pone.0223750.s008.docx]

**[
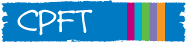
](http://www.google.co.uk/url?sa=i&rct=j&q=cpft&source=images&cd=&cad=rja&docid=PYeM1Y_IbH9T5M&tbnid=hA0lvO7q3F-SBM:&ved=0CAUQjRw&url=http://www.psychiatry.cam.ac.uk/dids/&ei=aRX5UevSB8yp0AXVj4HAAg&bvm=bv.49967636,d.d2k&psig=AFQjCNHC7XZAcj7xDnhSsSj19_cz5670kQ&ust=1375364835565506)**
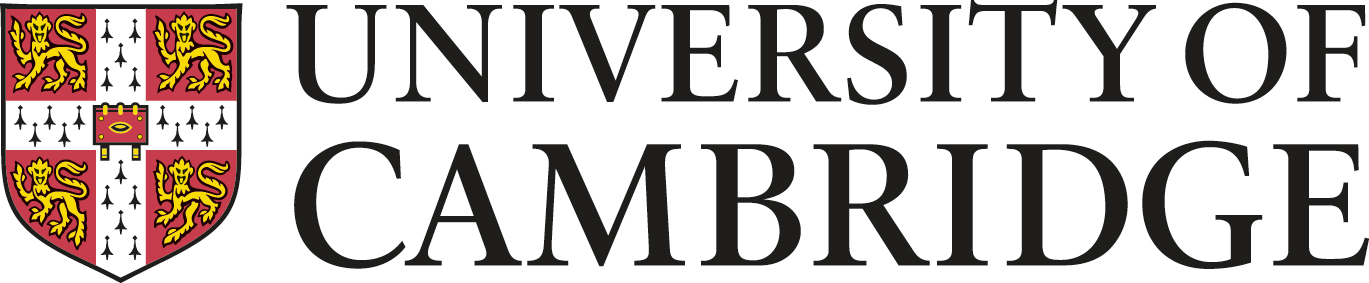


***Department of Psychiatry***

**Proof of concept study of vagus nerve stimulation using an external device for the treatment of behaviour problems in people with neurodevelopmental disorders, specifically Prader Willi Syndrome.**

**Short title**

Proof of concept study of vagus nerve stimulation.

1. **Background and Rationale**

Since our initial pilot study using implanted stimulators (Manning et al., 2015), externally worn vagus nerve stimulators are now available and for this reason, and given advice from scientific grant reviewers alongside the findings of the previous pilot study, a further small series of case studies are proposed. If the findings justify it, a larger study may then be indicated.

Prader-Will syndrome (PWS) is a genetically determined neurodevelopmental disorder characterised from childhood by excessive eating, severe obesity, sex and growth hormone deficiencies, intellectual disabilities, and problem behaviours (e.g. excessive repetitive and ritualistic behaviours, mild irritability and verbal and physical outbursts, which can sometimes last for hours). In the earlier pilot study, it was found that vagus nerve stimulation (VNS) was safe and acceptable, but that any effects on the overeating behaviour were equivocal. However, in two of the three participants who had a previous history of problem behaviours, there were unexpected and marked improvements in these behaviours reported by the participants and their families (Manning et al., 2015). As the pilot study had been designed to investigate the eating behaviour, we did not have longitudinal data on the rates of these behaviours over time. Given this observation, a further trial is now proposed with the primary aim of determining whether VNS, now from an externally worn medical device using an established stimulus protocol, is associated with a significant difference in the number and severity of problem behaviours.

These behaviours have a significant effect on the level of independence and quality of life of those with PWS and impact also on those who support them (Mazaheri et al., 2013). The UK PWS Association repeatedly receive requests for help following placement breakdown and, in some cases, the fact that the criminal justice system has become involved because of the behaviour. In over 800 queries received by the PWS Association in 2013, 25% of them were about severe problem behaviours (information from Jackie Waters, UK PWSA). In the absence of these outbursts support ratios could be reduced and the person with PWS could be more independent. This novel intervention, if effective, will enable people with PWS to live a more independent life without concerns that minor environmental triggers will result in episodes of severe problem behaviour which then escalate to prolonged outbursts associated with verbal or physical aggression. We predict that such treatment would significantly decrease health and social care costs and improve quality of life for people with PWS and those who support them.

Since the unexpected findings from the earlier pilot study in PWS, a review of the literature (see Manning et al., 2015) has found that there are reports of behavioural improvements where VNS has been used to treat epilepsy. Improvements in wellbeing have also been reported in people with autism spectrum conditions. These reports, however, are complicated by improvements, for example, in seizure control or mood. In addition to these observations the work of Porges and colleagues (the polyvagal theory; Porges & Furman, 2011) provides a useful explanatory model for why VNS might result in improvements in behaviour in people with neurodevelopmental syndromes where problem behaviours arise in the context of high arousal. It is for these reasons that we propose to extend the study beyond PWS.

**2 Objectives**

The primary objective is to determine whether vagus nerve stimulation (VNS) using an external device and established protocols, is associated with significantly fewer and less severe episodes of informant recorded outbursts, compared to no stimulation, thereby providing further evidence for its approval and use in the treatment of problem behaviours in people with PWS or with other neurodevelopmental disorders.

Additionally, there are a number of secondary objectives:

- To establish the percentage in each group who respond positively to vagus nerve stimulation with a clinically significant reduction of 50% or more and identify cognitive, autonomic and neural biomarkers associated with a positive response.
- In addition to collecting evidence on efficacy, to investigate safety and acceptability of the treatment in order to inform further studies.
- To investigate the characteristics of the behaviours that improve and the putative autonomic and neural mechanisms that are likely to mediate any reduction in such behaviours, in order to better understand the circumstances whereby the use of VNS might be generalized to treating problem behaviours more widely.

**3 Endpoints**

The primary endpoint is the measurement of the number and severity of informant recorded outbursts, as operationally defined. Secondary endpoints include the completion of specific questionnaires (detailed below) and the investigation of biomarkers associated with positive behavioural outcomes. The study will identify the percentage of responders, defined as those with clinically significant reductions of maladaptive behaviour of 50% or greater.

**4 Study Population**

**4.1 Study participants.**

6 individuals with PWS and 6 individuals with another neurodevelopmental disorder will be recruited to the study.

*4.1.1 Inclusion criteria*

- Male or female aged over 18 years of age.
- Genetically and clinically determined diagnosis of PWS or meeting clinical or the presence of another neurodevelopmental syndrome such as an autistic spectrum condition.
- History of problem behaviours of, on average, at least one significant informant-reported episode each week.
- Capacity to consent.
- Able to commit to the study duration and to attend assessments in Cambridge.

*4.1.2 Exclusion criteria*

- Meet exclusion criteria for MRI scanning and/or unable to tolerate MRI environment.
- Serious co-morbid physical or psychiatric disorder which would disrupt ability to comply with study demands (e.g. a history of serious bipolar disorder; sleep apnoea not well-controlled with CPAP; insulin dependent diabetes).
- Current or past history of neurological disorders or trauma, including epilepsy, and head injury.
- Currently or recently (within 12 months) participating in a clinical trial of an investigational medicinal product (CTIMP) or another medical device.
- Lacking the capacity to consent.

**5 Recruitment**

For all potential participants, no personal information about potential participants will be available to the research team until such point as the potential participant or his/her representative contacts the research team to make an expression of interest. This contact may be via the response slip in the information pack given to individuals identified either through their clinicians or through PWS supported living, providing the contact details of the interested participant (name, address, telephone) and nominating a ‘person who knows him/her best’ who can be contacted to arrange a date a for the initial visit, or through contacting the research team using the contact details available on an advertisement for the study.

**5.1 Recruitment of participants with PWS**

Potential participants with PWS will be identified through one of 3 routes:

1. Through self ­identification in response to an advertisement, including on the website and in the newsletter of the Prader-­Willi Syndrome Association UK (PWSA UK). Interested individuals will be able to contact the research team who will send out an information pack containing an information sheet for both the potential participant and his/her carer, a reply slip and a prepaid envelope.

2. Through managers of PWS­ specific supported living settings or other residential settings supporting one or more individuals with PWS. The research team will contact the managers of the settings to discuss the study and potential for residents to participate. With the agreement of managers, information packs will be sent to the setting and distributed to residents they know to be aged 18 and above and likely to have the capacity to consent.

3. Via NHS trusts. This may be through identification via Cambridgeshire and Peterborough Foundation Trust clinicians, other NHS/learning disability practitioners/services as Participant Identification Centres (PICs). Information packs would be given by the identifying clinician, with reply slips for interested participants to contact the research team.

**5.1 Recruitment of participants with other neurodevelopmental disorders**

Potential participants with other neurodevelopmental disorders will be identified in the following ways:

1. Through clinicians in the local specialist services for people with LD. In each case, clinicians will be asked to pass on information packs to those who may be appropriate for this study. The potential participant, or those who are supporting him/her, can then contact the research team. This contact will be followed-up by a visit.

2. Through organisations that provide support to people with LD and challenging behaviour. There are both NHS and independent sector services that specialise in the support of people with LD and challenging behaviour. The senior manager/clinician in these services will be informed about the study and if appropriate be asked to pass on information packs to potential participants. The same procedure will then be followed as above.

**6 Informed consent**

When the research team receive reply slips, they will contact the participant or his/her carer, depending on whose contact details the participant has chosen to provide, to arrange an initial visit to the participants home. It is at this visit that the researcher will go through the easy read information photograph booklet, watch the film with the participant and ensure participant is suitable for the study.

If the potential participant thinks they may still want to take part following this initial home visit, an opportunity to visit the Clinical Research Facility at Addenbrooke’s Hospital in Cambridge will be arranged. This will enable the potential participant, as well as their accompanying parent or carer, to see what the travel demands are like and to get a better understanding of what will happen if he/she chooses to participate. There will also be an opportunity to try VNS to see what it feels like.

Written informed consent will only be taken once this visit to Cambridge is complete, with the researcher, who will have completed training in informed consent and in the Mental Capacity Act. To ensure capacity to consent, the individual will be asked questions to assess his/her understanding and retention of the study details, their feelings about the study and ensure that their decision is consistent with these. This is in accordance with the assessment of capacity as described in Section 3 of the Mental Capacity Act 2005. Professor Holland and Dr Ring are both learning disability consultant psychiatrists with extensive experience working with people with PWS, Professor Holland is Chair in Learning Disabilities at the University of Cambridge and has expertise in gaining informed consent in this population. Katie Manning (research associate) is also experienced at assessing capacity and gaining consent in research with individuals with intellectual disabilities.

**6 Study design, procedures and assessment.**

**6.1 Study design and assessments**

The study takes the form of a longitudinal single case cross-over design. The study cannot be blind as the stimulation is apparent to participants. After a baseline phase of between 4 to 6 months of data collection and during which participants will be asked to wear the stimulator according to established protocols (four hours a day continuously or broken into 1 hour periods) but without it being activated, there will be an active treatment phase over a similar period of time. Towards the end of the active phase, there will be a period of reduction in the time the stimulator is worn and then no further stimulation.

The stimulus protocol will be that used for treatment of epilepsy using established protocols. For these externally worn devices the stimulator is worn for at least 4 hours a day – this can be broken into segments of time but should avoid be used during sleep. The stimulus strength used is that which is just detectable by the participant. The stimulation is continuous over the period that the device is worn. The stimulator consists of a pack similar to a mobile phone, a lead that the connects the pack with the electrode, and an electrode that is worn next to a specific place close to the ear so that it can stimulate through the skin a branch of the vagus nerve. The exact amount of time that participants are requested to wear the device will depend on early findings but it will always remain within established and accepted protocols for the treatment of epilepsy. It is anticipated that at the end of the active treatment phase, if benefits are found, rather than immediately stopping wearing the device for 4 hours a day it will be reduced to 2 hours a day and after some time stopped altogether. This will enable us to determine whether or not a similar level of benefit can be obtained at a lower dose of stimulation.

The primary outcome measures are the number and severity of informant-reported problem behaviours operationally defined and recorded using participant and observer diaries. This method of longitudinal data collection has been validated by Leah Bull for her completed PhD supervised by Prof Chris Oliver, a member of the research team. This data will be collated on a monthly basis by a research assistant.

Secondary behavioural outcomes will include: a) the participants response to a specified challenge using a methodology developed by Prof Oliver, filmed and subsequently rated blind for emotional and behavioural changes by an independent rater thereby overcoming the problem of lack of blindness and also allowing some measure of changes in resilience to a standard challenge; b) at the beginning, middle and end of each phase interviews of informants will be undertaken using valid and reliable questionnaires including the Challenging Behaviour Interview (Oliver et al., 2003), Behaviour Problems Inventory (Rojahn et al., 2001) and the Aberrant Behaviour Checklist, (Aman et al., 1985); the Repetitive Behaviour Questionnaire (Honey et al., 2012); the Life Experiences Checklist (Ager, 1990), the Personal Wellbeing Index (Cummins & Lau, 2005), and the Modified Carer Strain Index (Robinson, 1983). Also at the end of each phase a semi-structured interviews with participants and key informants will be undertaken to probe about any observed subtle changes in behaviours. These will be recorded and transcribed and rated against defined criteria blind to treatment status.

In order to better understand what predicts a positive behavioural response, we will explore possible psychological and neural brain biomarkers by undertaking the following assessments at similar time points on three occasions in both the non-stimulation and stimulation phases: the Mood, Interests and Pleasure Questionnaire (Ross & Oliver, 2003); tests of attention and set shifting using go/no go tasks established for use by people with PWS (Woodcock et al 2009, 2010); measures of cortical activation patterns and network activity using resting state and task-based fMRI (developed as part of a PhD project). Blood samples will be taken to measure glucose, insulin, and HbA1c at regular intervals as well as a one-off sample for diagnosis of PWS subtype in those with PWS for whom this is not already documented. Salival cortisol measures will also be recorded across the course of four separate days during the course of study (four in the hour after waking, one at noon and one at 8pm).

Heart rate variability will be derived from ECG and respiration measured using an Intelesens (Belfast) 3-axis ‘Zensor’ wearable monitor. For each participant ECG recording will take place in 24-hour blocks, (which we (Bhatoa and Ring 2015) have previously observed to be acceptable to adults with intellectual disability and their carers), with such blocks being recorded monthly during the sham and active conditions. Data will be stored on an integral SD card for subsequent off-line analysis. HRV will be determined from R-R intervals as root mean of squared successive differences and average HRV will be determined for each participant for brief (15 minutes) and prolonged (24 hours) periods.

Sleep monitoring will also be regularly undertaken, due to the association of increased risk of sleep apnoea in both VNS and PWS, using as Somnoscreen TM plus RC system (SOMNOmedics, Germany). This uses a three electrode ECG, pulse oximeter and movement sensors to record heart rate, blood oxygen desaturations and sleeping position throughout the night.

**6.2 Procedures**

In both the baseline and active phases, we will visit participants at home approximately monthly. At the home visit, we will conduct the questionnaires with participants and/or their carers, as appropriate, and will conduct the semi-stuctured interview if it is the end of a phase. We will also ask participants to do the cognitive tasks at some of these visits.

Throughout the study, parents or carers will fill out a daily diary entry on a form developed for this study, recording the participants behaviour during that day. These will be collated at the monthly home visit.

The participant will be asked to wear the ambulatory physiological monitoring device (monitoring heart rate and respiration) for one week at a time, four times during the study – two in each phase. This can be worn while carrying out regular daily activities at home, and can be removed and replaced easily for washing.

Participants will also be asked to provide saliva samples over the course of a single day on four occasions in the study, to measure cortisol levels. This will be done using a swab which is masticated for approximately two minutes before being return to a tube. These are stable at room temperature and can be returned via post.

On four occasions – twice in each phase –we will ask the participant to come to visit us in Cambridge. Participants travel to and from Cambridge will be paid for, as will that of an accompanying parent/carer, as well as food and drink while in Cambridge. Participants will come to the Wellcome Trust Clinical Research Facility (CRF) at Addenbrooke’s hospital in Cambridge, where they will stay overnight. At these visits, we will check the VNS settings, monitor their sleep using the Somnoscreen system, take the blood samples and carry out the fMRI scan. fMRI scans will be done at the Wolfson Brain Imaging Clinic, which is also located on the Addenbrooke’s site. Scans will include both resting-state and task-based fMRI, with the cognitive tests of attention and set-shifting completed in the scanner. While in Cambridge we will also carry out the videorecorded behavioural challenge test.

**7 Data analysis**

**7.1 Outcome measures**

The primary outcome measures are the number and severity of operationally defined outbursts as measured using participant and informant diaries.

Secondary behavioural outcomes will include:

1. During each treatment phase the participant's response to a specified challenge using a methodology developed by CO, filmed and subsequently rated blind for emotional and behavioural changes by an independent rater, thereby overcoming potential threats to blindness given that participants will be aware of active VNS
2. Percentage of responders with a clinically significant reduction of 50% or more in the rate of problem behaviours.
3. 3. Scores on specific valid and reliable rating scales repeated over time including the Challenging Behaviour Checklist, Behaviour Problems Inventory, Aberrant Behaviour Checklist, Repetitive Behaviour Questionnaire, and Life Exepeiences Checklist.
4. 4. The identification of cognitive, autonomic and neural biomarkers associated with positive behavioural outcomes.
5. 5. Findings from qualitative interviews of family or support staff.

**7.2 Planned analyses**

Longitudinal data obtained will be analyzed using established methods with cases as their own controls (see Perdices & Tate, 2009; de Vries & Morey, 2013). Data will be plotted out, visualized, and statistically analyzed using polynomial regression identify the ‘turning point’ in the treatment phase when behavior is observed to improve and establishing whether all those who improve do so at a similar time after treatment has started and in a similar way. Within the time constraints of the project, the point in time after which treatment was started past when improvement, if it hasn’t already occurred, is unlikely will be identified. Differences on behavioral measures in baseline and treatment conditions will be compared using Chi squared analyses giving confidence limits.

FMRI data will be analysed using both 1^st^ and 2^nd^ level general linear model (GLM) analyses to compare within cases and across time points. Network analyses of functional connectivity may also be appropriate.

**8 Data management**

**8.1 Data handling.**

Participants will be allocated an ID code on entry into the study and all data that is kept will only be attributable to that person through the code. The key to the code will be kept separately in a locked filing cabinet. Information on databases will be coded and access to the system will be password protected.

The analysis of fMRI data will take place via a secure network which allows a secure tunnel to be created into the WBIC server system from a remote computer by each researcher to access and analyse only their own project data. The remote computer used will be a university laptop.

Personal telephone numbers of potential participants will be used, once provided by interested potential participants, to contact the participant or his/her carer to arrange the initial visit. This information along with personal addresses, postcodes, emails and faxes will be kept in a locked filing cabinet.

All data transferred will be done in accordance to the NHS Code of Practice on Confidentiality and local Trust data protection policies and procedures using appropriate encryption.

The participants GP will be notified of their involvement in the study, and of the results of blood tests carried out in the course of their participation, as well as if we were to find. Participants will endorse a clause on the consent form confirming their permission for us to contact their GP for these purposes.

We will be sharing anonymised data with collaborators outside of the study team as part of open access as agreed by the funders and sponsor.

Only anonymised data will be analysed on Departmental computing facilities. Anonymised electronic data, including data entered into the computer from study measures and questionnaires, would be stored with reference only to the participant identifier, not their name.

**8.2 Data storage**

All electronic data are stored and backed up daily and secured on a database hosted at the School of Clinical Medicine, University of Cambridge.

The project will make use of Cambridge Clinical Trials Unit (CCTU) Data Management Service. This is a managed service for researchers where all aspects of data management, i.e. from design of data capture tool to data release for analysis will be catered. Data will be stored, managed and cleaned, as well as archived after project completion. The data backup services is in accordance to the security policy for Secure Data Hosting Service 2014 of the School of Clinical Medicine. Source data will be stored securely in the Principal Investigator’s (PI) laboratory (for a minimum of 10 years).

All identifiable data will be securely sent for central secure storage and stored in compliance with the Data Protection Act on University computers and approved by the sponsor. Permission for access will be granted only on a need ­to ­know basis to selected members of the study team. This is explained in the Information Sheets and included in the Informed Consent Forms. This will also be explained verbally when participants attend on the first visit with the opportunity to ask as many questions as they need.

Paperwork containing personal information (including completed questionnaires and study measures) will be stored in a locked filing cabinet at the research office. These will be marked with the participant identifier, not their names. Consent forms, which will contain participant names, will be stored in a separate locked drawer.

**9 References**

Ager, A. K. (1990). *The Life Experiences Checklist*. Windsor: NFER-Nelson.

Aman, M. G., Singh, N. N., Stewart, A. W., & Field, C. J. (1985). The aberrant behavior checklist: a behavior rating scale for the assessment of treatment effects. *American Journal of Mental Deficiency, 89*, 485-491.

Cummins, R.A. & Lau, A. (2005). Personal Wellbeing Index – Intellectual Disability. 3^rd^ Edition. Victoria, Australia: School of Psychology, Deakin University

de Vries, R.M., Morey, R.D. (2013). Bayesian hypothesis testing for single-subject designs. *Psychological Methods, 18*, 165-185.

Honey, E., McConachie, H., Turner, M. & Rodgers, J., (2012) Validation of the repetitive behaviour questionnaire for use with children with autism spectrum disorders. Research in Autism Spectrum Disorders, *6,* 355-364.

Manning, K. E., McAllister, C. J., Ring, H. A., Finer, N., Kelly, C. L., Sylvester, K. P., ... Holland, A. J. (2015). Novel insights into maladaptive behaviours in Prader-Willi syndrome: serendipitous findings from an open trial of vagus nerve stimulation. *Journal of Intellectual Disability Research.* Online first: 27 May 2015.

Mazaheri, M. M., Rae-Seebach, R. D., Preston, H. E., Schmidt, M., Kountz-Edwards, S., Field, N., Cassidy, S. & Packman, W. (2013). The impact of Prader–Willi syndrome on the family's quality of life and caregiving, and the unaffected siblings’ psychosocial adjustment. *Journal of Intellectual Disability Research*, *57,* 861–873.

Oliver, C., McClintock, K., Hall, S., Smith, M., Dagnan, D., & Stenfert-Kroese, B. (2003). Assessing the severity of challenging behaviour: psychometric properties of the Challenging Behaviour Interview. *Journal of Applied Research in Intellectual Disability, 16,* 53-61.

Perdices, M., & Tate, R.L. (2009). Single-subject designs as a tool for evidence-based clinical practice: are they unrecognised and undervalued? *Neuropsychological Rehabilitation, 19,* 904-927.

Porges, S. W., & Furman, S. A. (2011). The early development of the autonomic nervous system provides a neural platform for social behaviour: a polyvagal perspective. *Infant and Child Development, 20*, 106–118.

Robinson, B. C. (1983). Validation of a caregiver strain index. *Journal of Gerontology, 38*,344-348.

Rojahn, J., Polster, L.M., Mulick, J.A., & Wisniewski, J.J. (1989). Reliability of the Behaviour Problems Inventory. *Journal of the Multihandicapped Person, 2,* 283-293.

Ross, E. & Oliver, C. (2003). Preliminary analysis of the psychometric properties of the Mood, Interest and Pleasure Questionnaire (MIPW) for adults with severe and profound learning disabilities. *British Journal of Clinical Pyschology, 42,* 81-93.

Woodcock, K. A., Humphreys, G. W., Oliver, C., & Hansen, P. C. (2010). Neural correlates of task switching in paternal 15q11-q13 deletion Prader-Willi syndrome. *Brain Research, 1363*, 128-142.

Woodcock, K. A., Oliver, C., & Humphreys, G, W. (2009). Task switching deficits and repetitive behaviour in genetic neurodevelopmental disorders: Data from children with Prader-Willi syndrome chromosome 15 q11-q13 deletion and boys with Fragile-X syndrome. *Cognitive Neuropsychology, 26,* 172-194.
